# Genome-Wide Identification of VQ Motif-Containing Proteins and their Expression Profiles Under Abiotic Stresses in Maize

**DOI:** 10.3389/fpls.2015.01177

**Published:** 2016-01-05

**Authors:** Weibin Song, Haiming Zhao, Xiangbo Zhang, Lei Lei, Jinsheng Lai

**Affiliations:** State Key Laboratory of Agrobiotechnology and National Maize Improvement Center of China, Department of Plant Genetics and Breeding, China Agricultural UniversityBeijing, China

**Keywords:** maize, VQ domain-containing protein, drought stress, transcription factor, *WRKY* gene

## Abstract

VQ motif-containing proteins play crucial roles in abiotic stress responses in plants. Recent studies have shown that some VQ proteins physically interact with WRKY transcription factors to activate downstream genes. In the present study, we identified and characterized genes encoding VQ motif-containing proteins using the most recent version of the maize genome sequence. In total, 61*VQ* genes were identified. In a cluster analysis, these genes clustered into nine groups together with their homologous genes in rice and *Arabidopsis*. Most of the *VQ* genes (57 out of 61 numbers) identified in maize were found to be single-copy genes. Analyses of RNA-seq data obtained using seedlings under long-term drought treatment showed that the expression levels of most *ZmVQ* genes (41 out of 61 members) changed during the drought stress response. Quantitative real-time PCR analyses showed that most of the *ZmVQ* genes were responsive to NaCl treatment. Also, approximately half of the *ZmVQ* genes were co-expressed with *ZmWRKY* genes. The identification of these *VQ* genes in the maize genome and knowledge of their expression profiles under drought and osmotic stresses will provide a solid foundation for exploring their specific functions in the abiotic stress responses of maize.

## Introduction

Maize is one of the most important crops worldwide, and has recently become the most widely planted crop in China. Like any other crop, maize is greatly affected by various abiotic stresses such as drought. Generally, plants respond to abiotic stresses through several different strategies such as avoidance and tolerance. Many stress-related genes have been isolated from maize and other plants. Among these stress-related genes are those coding transcription factors that are involved in multiple stress response pathways. Some of the best-characterized stress-responsive transcription factors are C-repeat-binding factor (CBF)/dehydration-responsive element-binding (DREB) (Liu et al., 2013), NF-YB1 (Nelson et al., 2007), NAC (Hu et al., 2006), bZIP (Zhang et al., 2015), and WRKY proteins (Hu et al., 2013). Among these transcription factors, WRKYs are encoded by one of the largest gene families involved in abiotic stress responses (Eulgem et al., 2000; Dong et al., 2003; Chen et al., 2012). Genes encoding WRKY transcription factors have been identified in many species including maize, rice and *Arabidopsis* (Rushton et al., 2010; Wei et al., 2012). Members of the *WRKY* gene family have been shown to participate in several biotic and abiotic stress responses (Jiang and Deyholos, 2009; Li et al., 2009, 2013), leaf senescence (Robatzek and Somssich, 2002; Miao et al., 2004), hormone signaling (Chen et al., 2010; Shang et al., 2010), and seed development (Luo et al., 2005).

Several studies have shown that VQ domain-containing proteins can physically interact with WRKY transcription factors (Chi et al., 2013; Lin and Jing, 2015). The VQ proteins contain 50–60 conserved amino acids in the VQ (FxxxVQxLTG) motif, and interact with WRKY transcription factors via the conserved residues V and Q (Lai et al., 2011). AtVQ9 was reported to act antagonistically with WRKY8, which directly bind to the promoter of *RDA29A* in response to salt stress. Transgenic *Arabidopsis* plants with reduced AtVQ9 expression or overexpressing WRKY8 showed tolerance to osmotic stress (Hu et al., 2013). Two other VQ domain-containing proteins, SIB1 and SIB2, were shown to interact with WRKY33 by recognizing the C-terminal WRKY domain. The expression levels of SIB1 and SIB2 were up-regulated in response to the necrotrophic pathogen *Botrytis cinerea*. Overexpression of SIB1 in *Arabidopsis* showed enhanced resistance to *B.cinerea*. Further analyses indicated that the N-terminus of both SIB1 and SIB2 contained dual subcellular localization signals for the chloroplast and nucleus (Lai et al., 2011). MSK1, also known as AtVQ21, acts as a substrate of MAPK4 (MAP kinase 4), interacting directly with WRKY25 and WRKY33 to activate the MAPK4-regulated defense response (Andreasson et al., 2005). *AtCAMBP25*, which encodes another VQ motif-containing protein, was shown to function as a negative regulator in the osmotic stress response (Perruc et al., 2004). *AtVQ14* was shown to regulate endosperm growth through its interaction with *MINISEED3*, which encodes the WRKY transcription factor WRKY10 (Luo et al., 2005; Wang et al., 2010). A large-scale analysis of the interactions between *Arabidopsis* VQ and WRKY proteins in yeast cells showed that VQ proteins could act as cofactors for group I and IIc WRKY proteins (Cheng et al., 2012). VQ motif-containing proteins could also partner with other transcription factors such as PIF, a bHLH-type transcription factor (Li et al., 2014c).

In recent years, 34, 40, and 74 *VQ* family genes have been identified in *Arabidopsis* (Cheng et al., 2012), rice (Kim et al., 2013; Li et al., 2014b), and soybean (Wang et al., 2014), respectively. More recently, 18 *VQ* genes were identified in grapevine (*Vitis vinifera* L.) (Wang et al., 2015). The expression profiles of *VQ* genes were analyzed in response to pathogen infection and drought stress in rice, and in response to low-nitrogen stress in soybean. However, the *VQ* genes in maize have remained largely uncharacterized, although large number of genomic and RNA-seq datasets are available for maize (Schnable et al., 2009; Lai et al., 2010; Jiao et al., 2012; Chen et al., 2014; Li et al., 2014a). In this study, we searched the maize genome to identify *VQ* genes identification. We conducted a systematic phylogenetic analysis comparing the maize *VQ* genes with those in rice and *Arabidopsis*. To infer the potential functions of these genes, we analyzed the expression profiles of *ZmVQ* genes using RNA-seq data and qRT-PCR obtained from maize seedling shoots under long-term drought stress and salt treatment, respectively. The identification of these *ZmVQ* genes and knowledge of their transcription patterns under abiotic stress will be useful for further studies on the molecular mechanisms of these important transcription factors in the abiotic stress tolerance of maize.

## Materials and Methods

### Searches for VQ Proteins in Maize Database and Phylogenetic Analysis

The VQ motif (Pfam05678) was used as a query to scan the B73 filter gene database. BLASTP searches of protein libraries^1^ were also performed using the full-length amino acid sequence predicted for each newly identified gene. Only predicted protein sequences containing VQ motifs were defined as ZmVQ proteins. Multiple sequence alignment of the 61 ZmVQ proteins was conducted using the MUSCLE method (Arsova et al., 2010; Grant et al., 2010; Shi et al., 2013; Li et al., 2015). Then, a phylogenetic tree was constructed using the neighbor-joining method with MEGA6 software and bootstrap analysis of 1,000 replicates.

### Analysis of Gene Transcription Patterns in Different Tissues Using Public Datasets

To analyze differences in transcriptional patterns among various tissues, the expression values of *ZmVQ* genes were downloaded from our previous work (Chen et al., 2014). The samples used in that work included seed tissues (embryo and endosperm), root, shoot, shoot apical meristem, leaf, cob, tassel, silk, ovule, and immature ear. The detailed descriptions of the 78 different tissues are summarized in **Supplementary Table S1**. The methods for normalizing transcript levels and constructing heat maps were described previously (Chen et al., 2014).

### Identification of *cis*-elements in Promoters of *ZmVQ* and *ZmWRKY* Genes

The distribution of seven *cis*-elements was determined by analyzing the promoter regions (2000-bp DNA sequences upstream of gene start codons) of *ZmVQ* and *ZmWRKY* genes. The *cis*-elements are binding sites for the following classes of transcription factors: WRKY transcription factor (W-box, TTGAC[C/T]) (Chen et al., 2012), drought response element (DRE, [A/G]CCGAC) (Sakuma et al., 2002), and calmodulin-binding transcription activator (CG-box, [A/C/G]CGCG[C/G/T]), all of which are involved in various abiotic stress responses (Yang and Poovaiah, 2002); MYB transcription factor, which is involved in drought inducibility (MBS, CAACTG) (Urao et al., 1993); abscisic acid responsive element (ABRE, [C/T]ACGTG[G/T]) (Osakabe et al., 2014), salicylic acid responsive element (SARE, TGACG) (Pieterse and Van Loon, 2004), and environmental stimuli responsive element (G-box, CACGTG) (Williams et al., 1992). The sequences of *ZmVQ* promoters were downloaded from the Phytozome v.10.1.

### Plant Materials, Growth Conditions and Drought Stress Treatment

Seeds of the maize inbred line B73 were germinated in an incubator, and then transferred to pots (10 cm diameter, 10 seedlings per pot) containing a vermiculite: soil mixture (1:1, v/v). The seedlings were grown under the following conditions: 25°C ± 2°C, 60–70% humidity, 18-h light/6-h dark photoperiod, with natural sunlight. Each plot had 10 seedlings. For the drought treatment, the pots containing seedlings were fully irrigated until seedlings reached the three-leaf stage. Then, the seedlings were subjected to drought stress by withholding water for 6 days. The seedlings were re-watered on day 6. The seedling shoots were sampled at three time points; day 3, 6, and 7 (24 h after re-watering), respectively. The controls were watered as necessary and sampled at the same time points. Three biological replicates were collected. All samples were wrapped in aluminum foil, frozen in liquid nitrogen, and stored at -80°C until use.

### Osmotic Stress Treatment

Seeds of the B73 inbred line were used in this experiment. Seeds from the middle of the ear were sterilized using H_2_O_2_ (30%), and washed three times with sterile water. Then, the seeds were transferred to filter paper moistened with sterile water on a plastic tray, and covered with two pieces of filter paper that were also moistened with sterile water. The seed trays were kept in an incubator at 28°C, 60% humidity, in the dark. After 2 days, germinated seeds were transplanted into pots (10-cm diameter, 10 seedlings per pot) containing a vermiculite:soil mixture (1:1, v/v). The seedlings were grown in the incubator at 28°C, 60% humidity, under an 18-h light/6-h dark photoperiod. RNA was isolated from 2-week-old seedlings subjected to a NaCl osmotic stress treatment. For the NaCl treatment, the pots containing seedlings were irrigated with 250 mM NaCl (Hu et al., 2013), while the controls were irrigated with water. Seedling shoots were collected at 0, 24, and 48 h after the treatment. The experiment was repeated three times, with three plants per repeat. All samples were wrapped in aluminum foil, frozen in liquid nitrogen, and stored at -80°C until use.

### Total RNA Extraction and Construction of Sequencing Libraries

Two biological replicates were used for RNA isolation, construction of libraries, and RNA sequencing. Total RNAs were isolated from maize seedling shoots using Trizol reagent (Invitrogen^2^) according to the manufacturer’s protocol. RNA-seq libraries were constructed as described previously (Chen et al., 2014). The libraries were sequenced using the Illumina HiSeq2000 platform^3^. After sequencing, raw reads were aligned to the B73 reference genome (v2) using Tophat 2.0.6 (Trapnell et al., 2009) with default settings for all parameters. In a correlation analysis, the average R^2^ between the two biological replicates was 0.94 (**Supplementary Figure S1**). The unique mapped reads were used to measure the transcript abundance of every gene using Cuﬄinks (Trapnell et al., 2012).

### qRT-PCR Analysis

Total RNAs were isolated from maize tissue with TRIzol reagent (Invitrogen). DNase treatment was performed on 4 μg total RNA using RQ1 RNase-free DNase (Promega^4^) prior to first-strand cDNA synthesis using M-MLV Reverse Transcriptase (Promega). Real-time quantitative RT-PCR was performed using SYBR^®^ Premix Ex TaqTM (Perfect Real Time, TaKaRa^5^) on an ABI7500 instrument (Applied Biosystems^6^). Each 20 μL PCR reaction mixture contained 10 μL 2× SYBR Green Premix, 2 μL 10-fold diluted cDNA product, 0.4 μL ROX Reference Dye II, and 0.4 μL forward and reverse primers. The PCRs were performed with a holding step at 95°C for 30 s, followed by 40 cycles of denaturation at 95°C for 30 s, annealing at 55–60°C for 30 s and extension at 72°C for 30 s. Quantitative assays were performed on each cDNA sample three times. Relative gene expression levels were calculated using the 2^-ΔΔCT^ method (Livak and Schmittgen, 2001). β-*Actin* mRNA levels were determined with specific primers to allow normalization of transcript levels among samples. **Supplementary Table S2** lists the primers used for qRT-PCR.

To validate the RNA-seq data, we isolated RNA from stored samples of drought-treated plants (days 3, 6, and 7-rewatered) and their corresponding controls and conducted qRT-PCR analyses. We evaluated the expression levels of *ZmVQ* genes in drought-treated plants and control plants of the same age in the qRT-PCR analyses to ensure that differences in gene expression were due to the treatment and not differences in the developmental stage of the samples. Seven different tissues were used for tissue-specific expression analysis in the B73 genetic background. Ear (7 cm), tassel (16 cm), seed [16 days after planting (DAP)], endosperm (16 DAP), and embryo (16 DAP) tissues were harvested from plants at the tasseling stage in the field in the summer of 2015. Root and leaf samples were obtained from 2-week-old seedlings that were grown in the laboratory under the same conditions as those described in the osmotic treatment section. All samples were wrapped in aluminum foil, frozen in liquid nitrogen, and stored at -80°C until use.

Data were analyzed by one-way ANOVA using Microsoft Excel software. The student’s *t*-test at a significance level of 0.05 was used to detect significant differences between the treatment and control values. All expression data were obtained from three biological repeats. Values shown in figures are means of three repeats with standard deviation (SD).

### Analyses of *ZmVQ* Expression Profiles and Co-expression with *ZmWRKY* Based on RNA-seq Data

The relative expression levels of *ZmVQ* genes were calculated using the inferred formula (Yuan et al., 2006), where Ctreatment is the expression level of genes under drought treatment and Ccontrol is the expression level of genes in the control. Then, the relative expression level (Crelative = Ctreatment - Ccontrol) of *ZmVQ* genes was plotted to show the expression profiles (**Figure 4**). We analyzed co-expression patterns of *ZmVQ* and *ZmWRKY* genes (**Supplementary Table S3**) using RNA-seq data obtained from plants under drought at three time points. Genes whose expression levels were positively correlated (Pearson Correlation Coefficient > 0.90; (Li et al., 2014c) with those of other genes were selected for map drawing using Cytoscape (Shannon et al., 2003).

## Results

### Maize VQ Domain-Containing Protein Family has 61 Members

**FIGURE 1:**
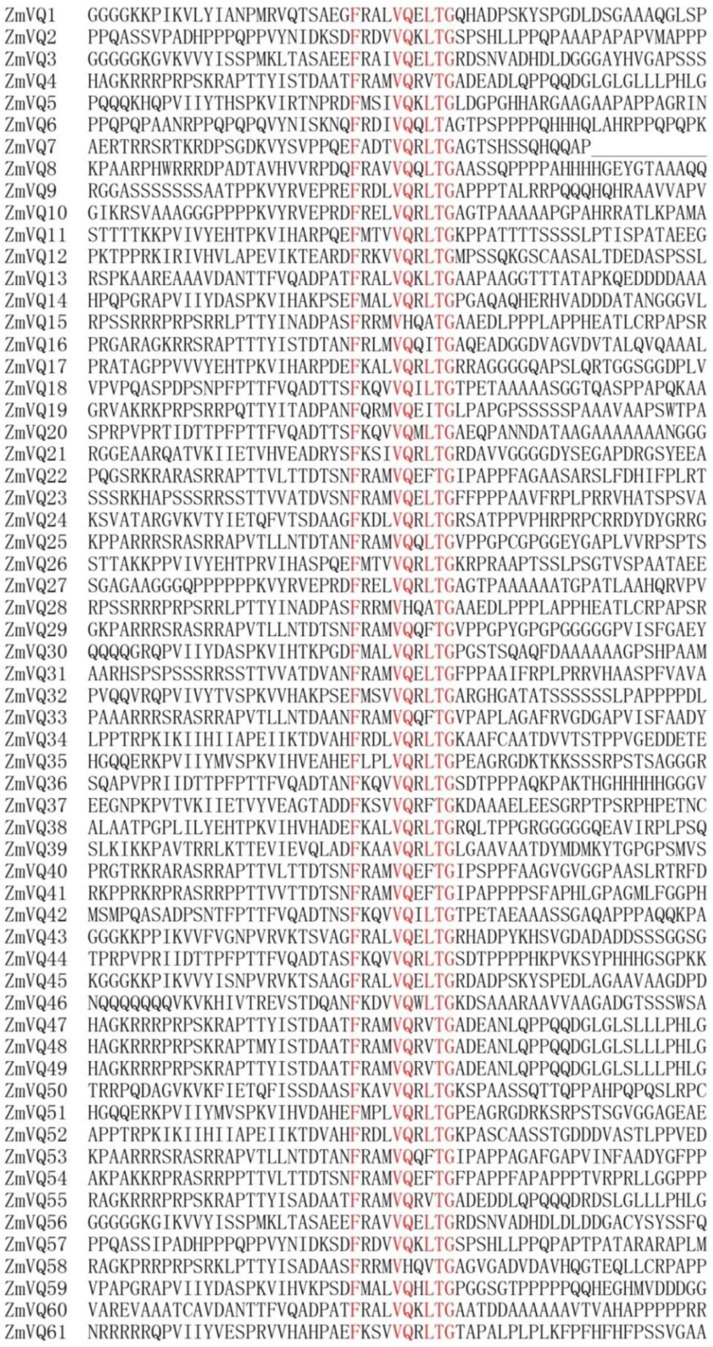
**Sequence alignment of 61 conserved VQ motif regions in maize.** Highly conserved amino acids in VQ motif are shown in red.

In total, we identified 61 VQ motif-containing proteins, which were numbered from ZmVQ1-ZmVQ61 based on the locations of their encoding genes on the chromosomes (**Table 1**; **Figure 1**; **Supplementary Figure S2**). Of the 61 proteins identified, 42 contained the conserved motif FxxxVQxLTG (42/61), and the other 19 contained FxxxVQxxTG or FxxxVQxLTx motifs (**Figure 1**). The core amino acids of three identified proteins (ZmVQ15, ZmVQ28, and ZmVQ58) were VH instead of VQ, similar to OsVQ37 and OsVQ39 in rice (Kim et al., 2013). Most of the ZmVQ proteins had fewer than 300 amino acid residues and five (ZmVQ6, ZmVQ14, ZmVQ22, ZmVQ32, and ZmVQ40) had more than 300. The predicted isoelectric points of the VQ proteins varied from 5.05 (ZmVQ52) to 11.72 (ZmVQ6). Gene structure analyses showed that 54 out of 61 *ZmVQ* genes lacked introns, while the other seven (*ZmVQ1*, *ZmVQ9*, *ZmVQ20*, *ZmVQ37*, *ZmVQ38*, *ZmVQ51*, and *ZmVQ57*) had only one or two introns. Most of the *ZmVQ* genes were single-copy genes in the B73 reference genome, except for *ZmVQ15*, *ZmVQ28*, *ZmVQ48*, and *ZmVQ49*, which had two or three copies. **Table 1** shows detailed information for the *ZmVQ* genes, including their accession number, copy number, chromosome location, number of amino acid residues, molecular weight, and conserved motifs.

**Table 1:** ZmVQ motif-containing genes in maize.

Gene name	Accessions	Chromosome	Position (bp)		Copy number	Intron number	Amino acids	PI	MW (kDa)	Conserved VQ-motif sequence
		
			Start	End						
ZmVQ1	GRMZM2G417835	chr1	14384994	14386612	1	1	189	6.79	20.5117	FRALVQELTG
ZmVQ2	GRMZM2G420357	chr1	19475469	19476633	1	0	245	11.14	25.3897	FRDVVQKLTG
ZmVQ3	GRMZM2G318652	chr1	51894857	51895988	1	0	168	9	17.1789	FRAIVQELTG
ZmVQ4	GRMZM2G128644	chr1	52252488	52253871	1	0	201	7.85	20.5732	FRAMVQRVTG
ZmVQ5	GRMZM2G174650	chr1	98163516	98164868	1	0	269	9.68	27.8542	FMSIVQKLTG
ZmVQ6	GRMZM2G158976	chr1	102948793	102950569	1	0	308	11.72	32.5699	FRDIVQQLTA
ZmVQ7	GRMZM2G421934	chr1	108121650	108121832	1	0	61	9.98	6.536	FADTVQRLTG
ZmVQ8	GRMZM2G420630	chr1	108265783	108266565	1	0	145	7.13	15.3235	FRAVVQQLTG
ZmVQ9	GRMZM2G059064	chr1	179720212	179720868	1	2	127	10.78	13.1287	FRDLVQRLTG
ZmVQ10	GRMZM2G118172	chr1	256796579	256797344	1	0	118	10.54	0.01054	FRELVQRLTG
ZmVQ11	GRMZM2G174210	chr1	286181853	286182602	1	0	171	10.63	17.524	FMTVVQRLTG
ZmVQ12	AC206638.3_FG007	chr2	3085080	3085499	1	0	139	6.9	14.8377	FRKVVQRLTG
ZmVQ13	GRMZM2G023921	chr2	5836317	5837195	1	0	195	9.41	20.6742	FRALVQKLTG
ZmVQ14	GRMZM2G369742	chr2	55036620	55038324	1	0	315	9.56	32.6818	FMALVQRLTG
ZmVQ15	GRMZM2G147443	chr2	148868258	148868925	2	0	192	10.22	20.2358	FRRMVHQATG
ZmVQ16	GRMZM2G101409	chr2	217684825	217685795	1	0	202	6.09	20.8743	FRLMVQQITG
ZmVQ17	GRMZM2G354123	chr2	234191571	234192146	1	0	191	11.27	19.7975	FKALVQRLTG
ZmVQ18	GRMZM2G055404	chr3	4722232	4723313	1	0	215	9.72	21.6507	FKQVVQILTG
ZmVQ19	GRMZM2G378442	chr3	45046974	45048075	1	0	249	8.38	24.8667	FQRMVQEITG
ZmVQ20	GRMZM2G314520	chr3	197382762	197384196	1	1	251	9.92	26.1273	FKQVVQMLTG
ZmVQ21	AC194056.3_FG008	chr3	213548727	213548999	1	0	90	6.72	9.5955	FKSIVQRLTG
ZmVQ22	GRMZM2G066599	chr4	76383639	76385196	1	0	346	7.79	35.352	FRAMVQEFTG
ZmVQ23	GRMZM2G322950	chr4	180649502	180650342	1	0	199	9.23	20.8331	FRAMVQELTG
ZmVQ24	GRMZM2G153597	chr4	225854653	225855069	1	0	131	8.62	14.2658	FKDLVQRLTG
ZmVQ25	GRMZM2G010333	chr4	235719536	235720135	1	0	191	8.71	19.2745	FRAMVQQLTG
ZmVQ26	GRMZM2G124290	chr5	4619463	4620282	1	0	182	10.63	18.4941	FMTVVQRLTG
ZmVQ27	GRMZM2G129140	chr5	13723734	13724402	1	0	121	9.81	12.0265	FRELVQRLTG
ZmVQ28	GRMZM2G325208	chr5	36298809	36299476	2	0	192	10.22	20.2358	FRRMVHQATG
ZmVQ29	AC207043.3_FG002	chr5	86012514	86014204	1	0	234	10.08	23.6371	FRAMVQQFTG
ZmVQ30	GRMZM2G346837	chr5	170825684	170827402	1	0	287	8.89	30.3402	FMALVQRLTG
ZmVQ31	GRMZM2G061941	chr5	208198242	208199181	1	0	208	8.77	20.9701	FRAMVQELTG
ZmVQ32	GRMZM2G003669	chr6	702374	703544	1	0	324	9.62	33.6895	FMSVVQRLTG
ZmVQ33	GRMZM2G420715	chr6	93586452	93587633	1	0	240	7.21	24.4229	FRAMVQQFTG
ZmVQ34	GRMZM2G082118	chr6	98257582	98258396	1	0	189	5.32	20.6533	FRDLVQRLTG
ZmVQ35	GRMZM2G099691	chr6	102547587	102548371	1	0	215	11.09	22.191	FLPLVQRLTG
ZmVQ36	GRMZM2G174558	chr6	160717320	160718691	1	0	217	10.13	22.9341	FKQVVQRLTG
ZmVQ37	GRMZM5G814101	chr6	165559690	165560640	1	1	109	8.79	12.0866	FKSVVQRFTG
ZmVQ38	GRMZM2G355499	chr7	6739000	6742999	1	1	210	10.52	21.6087	FKALVQRLTG
ZmVQ39	GRMZM2G083285	chr7	28262041	28262919	1	0	291	9.31	31.7463	FKAAVQRLTG
ZmVQ40	GRMZM2G126413	chr7	100523345	100525002	1	0	416	6.29	40.8146	FRAMVQEFTG
ZmVQ41	GRMZM2G316033	chr7	166745558	166746285	1	0	228	6.66	23.4719	FRAMVQEFTG
ZmVQ42	GRMZM2G151909	chr8	22843785	22844855	1	0	212	8.85	22.1573	FKQVVQILTG
ZmVQ43	GRMZM2G036980	chr8	120149428	120150492	1	0	156	5.42	15.6671	FRALVQELTG
ZmVQ44	GRMZM2G180668	chr8	123810520	123811466	1	0	220	10.06	23.1894	FKQVVQRLTG
ZmVQ45	AC203294.3_FG012	chr8	171193955	171194488	1	0	178	5.65	18.8565	FRALVQELTG
ZmVQ46	GRMZM5G800535	chr9	18534327	18534790	1	0	142	6.57	14.4858	FKDVVQWLTG
ZmVQ47	GRMZM2G374336	chr9	44338927	44339600	1	0	176	11.24	18.1407	FRAMVQRVTG
ZmVQ48	GRMZM5G849527	chr9	44340019	44340462	3	0	123	10.03	12.7896	FRAMVQRVTG
ZmVQ49	GRMZM5G864059	chr9	44341110	44341553	3	0	123	10.03	12.7896	FRAMVQRVTG
ZmVQ50	GRMZM2G138370	chr9	64622801	64623710	1	0	139	6.71	14.8997	FKAVVQRLTG
ZmVQ51	GRMZM2G069169	chr9	89016989	89017775	1	1	209	10.43	21.6114	FMPLVQRLTG
ZmVQ52	GRMZM2G122447	chr9	100304257	100305904	1	0	191	5.05	20.6691	FRDLVQRLTG
ZmVQ53	GRMZM2G333049	chr9	110319842	110320893	1	0	233	7.26	24.0476	FRAMVQQFTG
ZmVQ54	GRMZM2G035531	chr9	119969236	119970106	1	0	272	7.17	27.7747	FRAMVQEFTG
ZmVQ55	GRMZM2G014839	chr9	133482875	133483605	1	0	206	7.01	21.0485	FRAMVQRVTG
ZmVQ56	GRMZM2G129815	chr9	134088631	134089643	1	0	181	6.39	19.3031	FRAVVQELTG
ZmVQ57	GRMZM5G864133	chr9	149545721	149547672	1	1	236	11.14	24.4656	FRDVVQKLTG
ZmVQ58	GRMZM2G180262	chr10	2141505	2142516	1	0	219	7.08	22.2236	FRRMVHQVTG
ZmVQ59	GRMZM2G060720	chr10	115122727	115124234	1	0	300	7.21	30.5445	FMALVQHLTG
ZmVQ60	GRMZM2G064903	chr10	145199393	145200369	1	0	211	8.51	22.1278	FRALVQKLTG
ZmVQ61	GRMZM2G475276	chr10	148934513	148935055	1	0	178	9.91	18.9737	FKSVVQRLTG


### Phylogenetic Analysis of VQ Domain-Containing Genes in Maize

**FIGURE 2:**
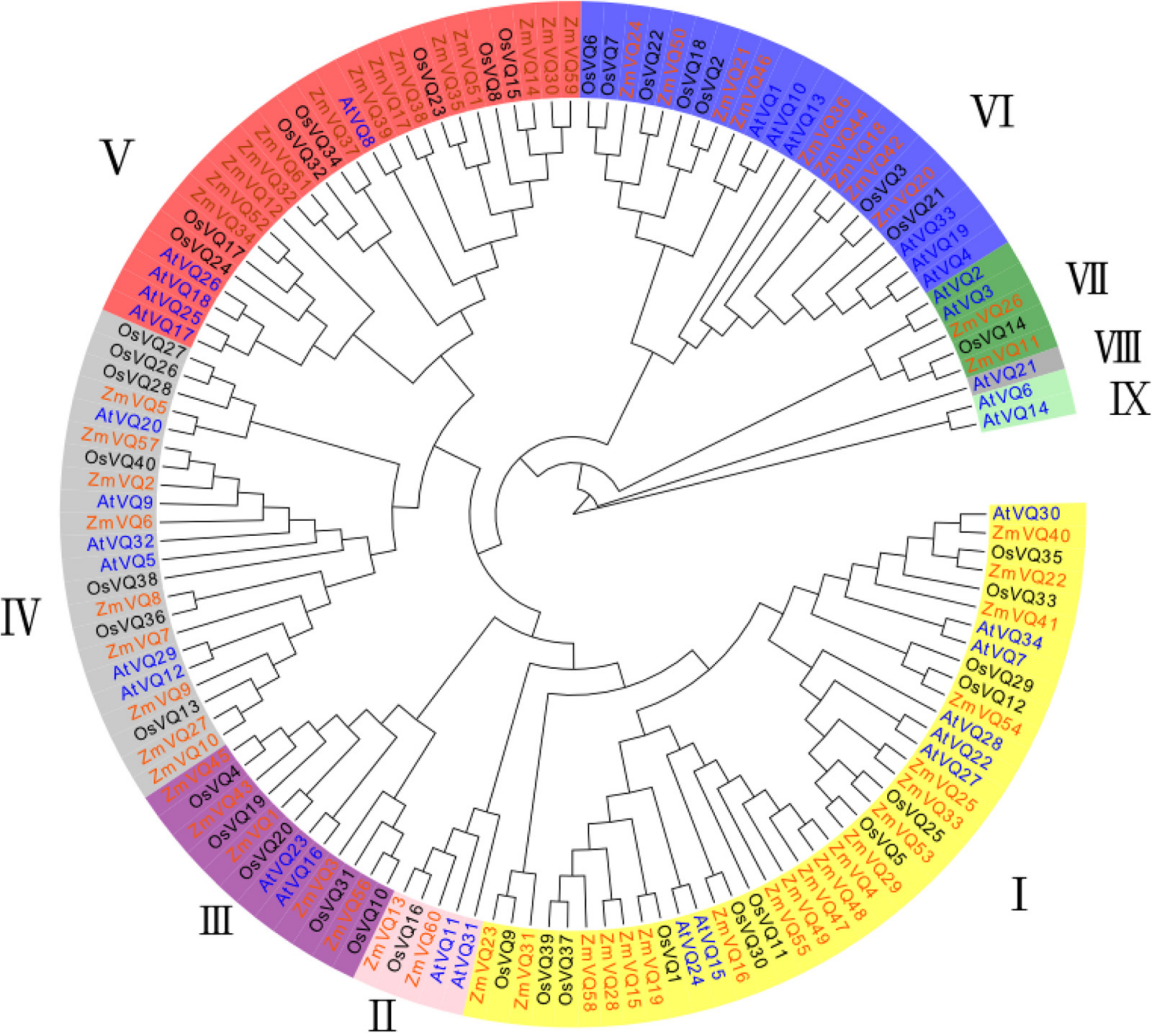
**Phylogenetic tree of *VQ* genes from maize, *Arabidopsis* and rice.** Sixty-one *ZmVQ* genes, 34 *AtVQ* genes, and 40 *OsVQ* genes clustered into nine groups (groups I–IX, represented by different colors).

To explore the relationships of ZmVQ genes in maize, we conducted a phylogenetic analysis of the *VQ* genes from maize, rice and *Arabidopsis*. This analysis was conducted using the neighbor-joining method and 61, 34, and 40 *VQ* genes from maize, *Arabidopsis* and rice, respectively. The *g*enes clustered into nine distinct groups based on the structural features of their protein sequences (ZmVQI–V; **Figure 2**). Among the nine groups, there were 20 *ZmVQ* genes in group I, 2 *ZmVQ* genes in group II, 5 *ZmVQ* genes in group III, 9 *ZmVQ* genes in group IV, 14 *ZmVQ* genes in group V, 9 *ZmVQ* genes in group VI, and 2 *ZmVQ* genes in group VII. Groups VIII or IX were largely rice-specific, with no maize *VQ* genes and only one *Arabidopsis VQ* gene (*AtVQ21*) in group VIII.

All of the VQ domain-containing proteins contained the conserved motif of FxxxVxxxTx (**Figure 1**). Among the 61 ZmVQ proteins, 45 had the amino acid “L” next to the “TG,” eight had the amino acid “F” (ZmVQ22, 29, 33, 37, 40, 41, 53, and 54), six had the amino acid “V”(ZmVQ4, 47, 48, 49, 55, and 58), two had the amino acid “I” (ZmVQ16 and 19), and two had the amino acid “A” (ZmVQ15 and 28). The proteins with an “L” in this conserved domain were distributed into among all nine groups, but those with the rarer residues (F, V, I, and A) were restricted to groups I and V. Rice had 11 VQ proteins with residues other than “L” next to the “TG”: “F” (OsVQ5, 12, 25, 29, 33, 34, and 35), “V” (OsVQ11, 30, and 37), “I” (OsVQ1), while *Arabidopsis* had seven: “F” (AtVQ7, 27, 28, 30), “V” (AtVQ15 and 24), and “Y” (AtVQ22). Those with rarer amino acids in this domain also clustered into groups I and V, similar to those in maize (**Figure 2**).

### Expression Patterns of 61 ZmVQ Genes in Different Maize Tissues

**FIGURE 3:**
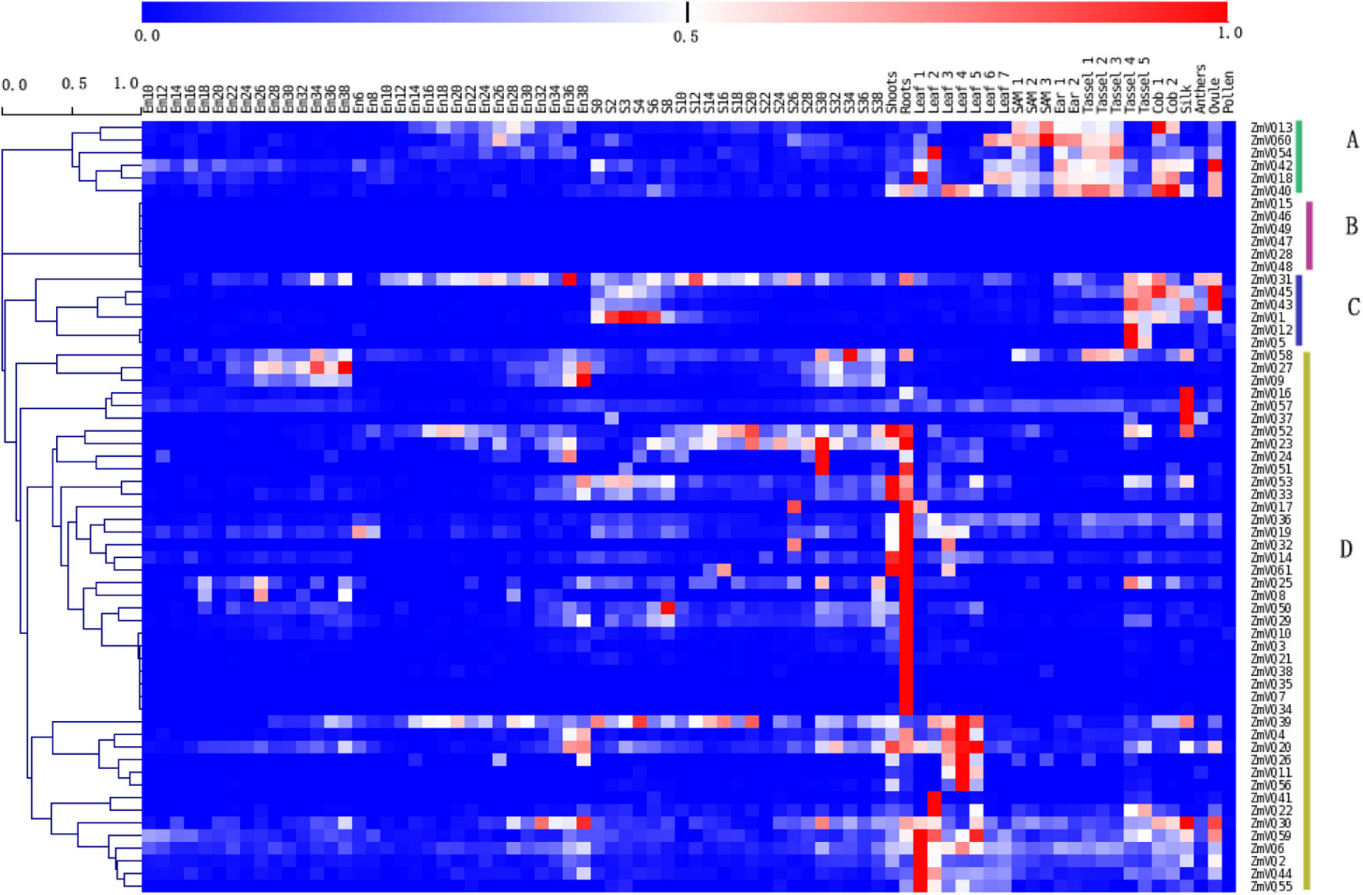
**Heat map showing expression levels of 61 ZmVQ genes in 78 different tissues, based on RNA-seq data.** Different colors in map represent gene transcript abundance values as shown in bar at top of figure.

The expression patterns of the *ZmVQ* genes were investigated using available RNA-seq datasets from 78 different tissues (**Supplementary Table S1**). The 61 ZmVQ genes were clearly divided into four groups based on their expression patterns. Groups A, B, and C each contained six *ZmVQ* genes, and group D contained 43 *ZmVQ* genes. Genes in groups A and C tended to be expressed at higher levels in reproductive tissues such as the cob, tassel, and ovule (**Figure 3**). For example, *ZmVQ13* and *ZmVQ40* showed the highest expression levels in the cob, and *ZmVQ42, ZmVQ43*, and *ZmVQ45* showed the highest expression levels in the ovule. Genes in group B showed no detectable expression in the RNA-seq libraries analyzed. Genes in group D were expressed at high levels in vegetative organs such as shoots, roots, and leaves. Some genes in group D showed tissue-specific expression patterns. For example, *ZmVQ3*, *7*, *10*, *21*, *34*, *35*, and *38* showed the highest expression levels in roots, while *ZmVQ2, 4, 6,11,20,22, 26, 41, 39, 44, 55, 56*, and *59* were expressed at higher levels in leaves. Transcripts of *ZmVQ41* were detected in leaf tissues, based on our RNA-seq data. The tissue-specific expression patterns of 12 *ZmVQ* genes were validated by qPCR analysis using seven different tissues (**Supplementary Figure S3**; **Supplementary Table S6**).

### Expression Patterns of ZmVQ Genes in Response to Drought Stress

**FIGURE 4:**
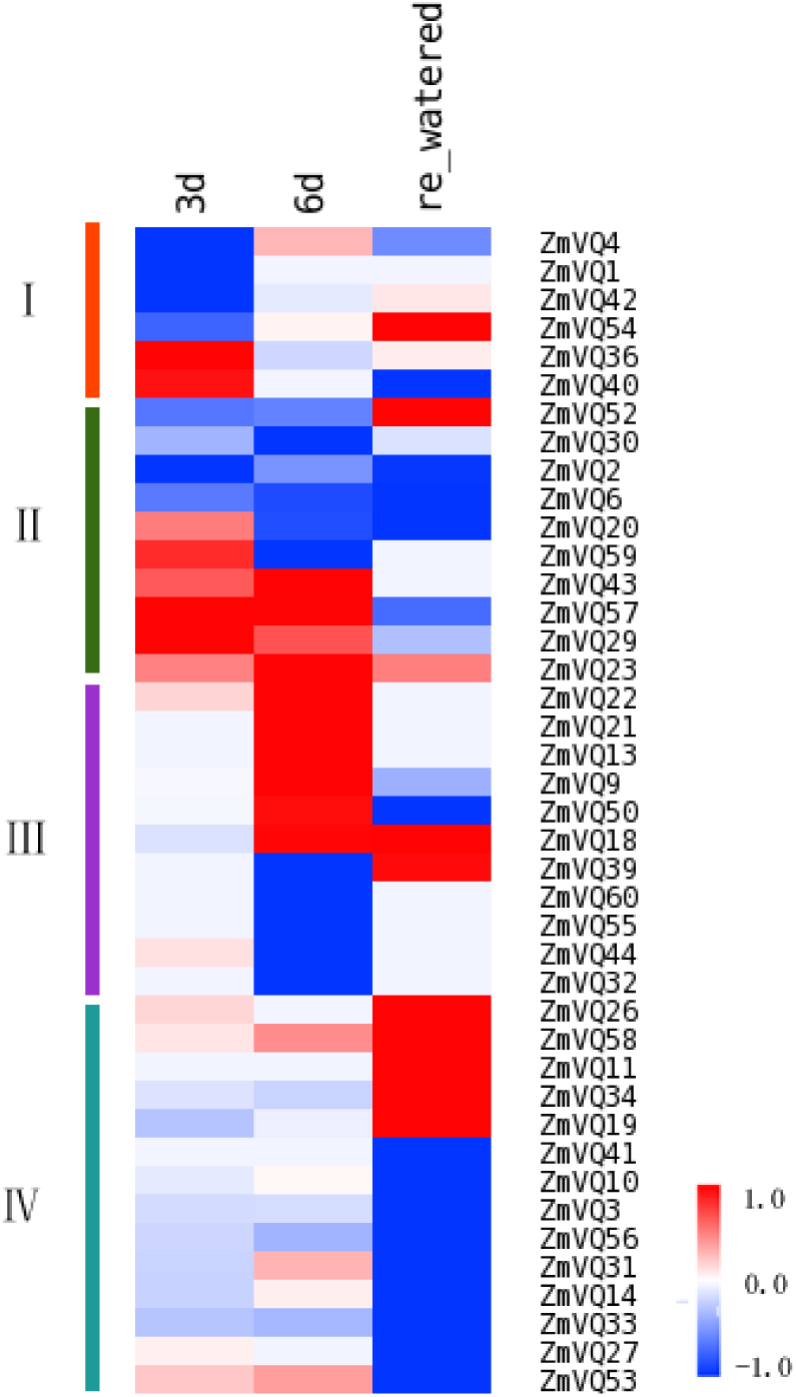
**Heat map showing ZmVQ expression patterns in maize under long-term drought stress.** *ZmVQ* gene expression levels were measured in tissues of seedlings at different stages of drought stress. Different colors in map represent gene transcript abundance values as shown in bar at top of figure. Color changes from blue to red in bar represent expression level changes from -1 to 1.

Previous studies have shown that some *VQ* genes in *Arabidopsis* and rice were induced by NaCl, dehydration, and drought, based on qRT-PCR analyses (Hu et al., 2013; Kim et al., 2013). We analyzed the expression patterns of *ZmVQ* genes under drought stress. Total RNA was extracted from the drought treated seedlings and libraries were constructed for sequencing. First, the transcript levels of the drought-responsive marker gene ZmNAC111 (Mao et al., 2015) were quantified to evaluate the effect of our drought treatment. In our RNA-seq data, the expression level of ZmNAC111 changed dramatically in plants under drought stress (FPKM values of 38.80 and 100.03 on days 3 and 6 of drought stress, respectively) and decreased markedly after re-watering (FPKM value of 5.43 on day 7, after re-watering). These results confirmed that our drought treatment method was effective. Under these conditions, 41 *ZmVQ* genes were drought-responsive. According to their different expression patterns under drought treatment, the 41 drought-responsive *ZmVQ* genes clustered into four groups (**Figure 4**). The first group contained six genes that showed early drought-responsive expression, with decreased (ZmVQ1, 4, 42, 54) or induced (ZmVQ36, 40) transcript levels after 3 days of drought stress. Then, four genes of ZmVQ1, 4, 42, 54 were transcriptional up-regulated slightly at time point of 6 days (**Figure 4**), while it was opposed for ZmVQ36 and ZmVQ40.The second group consisted of 10 genes showing changed expression levels at both 3 and 6 days of drought stress. The third group contained 11 genes that showed a slower response to drought stress, with increased or decreased transcript levels after 6 days of drought stress. Among them, *ZmVQ50* was expressed in response to re-watering. The fourth group consisted of 14 genes that did not show drought-responsive expression, but showed increased expression levels after re-watering. Together, our results showed that most of the *VQ* genes were responsive to drought stress, with complex expression patterns during drought stress and re-watering. The expression patterns of 10 *ZmVQ* genes were further validated by qRT-PCR (**Supplementary Figure S4**).

### Expression of *ZmVQ* Genes Under NaCl Osmotic Stress

**FIGURE 5:**
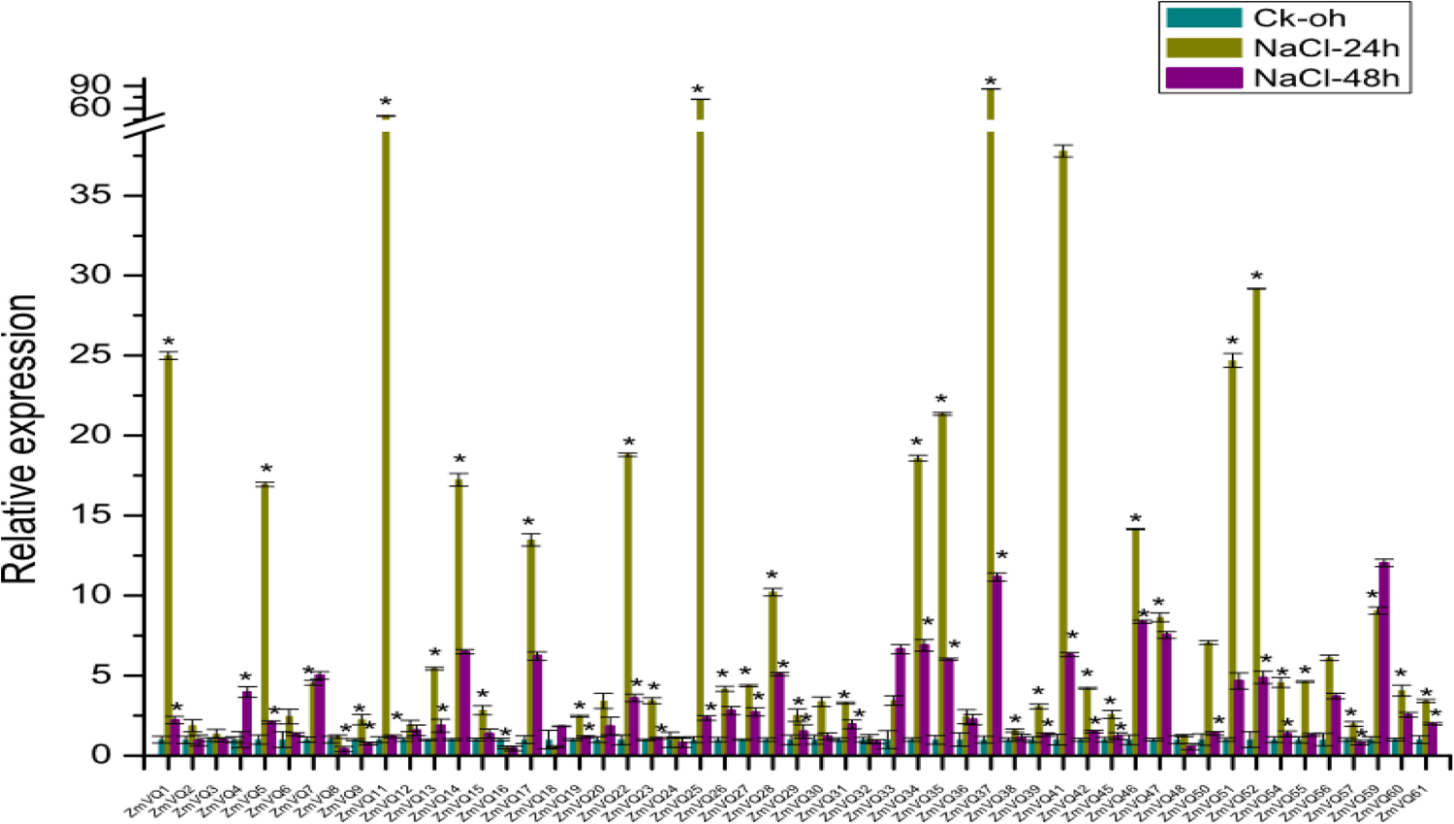
**Expression of 53 *ZmVQ* genes in response to NaCl treatment.** Gene expression in leaves of 2-week-old seedlings was analyzed by qRT-PCR. Expression levels in control (0 h) were normalized to 1. Asterisk indicates significant difference between treatment and control.

Hu et al. (2013) reported that *Arabidopsis VQ9* was responsive to osmotic stress (Hu et al., 2013). To study whether the maize *VQ* genes were also responsive to osmotic stress, we conducted qRT-PCR analyses of 53 *ZmVQ* genes, but not the eight *ZmVQ* genes for which there were no gene-specific PCR primers. Total RNA was isolated from maize seedlings under NaCl osmotic stress. The relative expression values of the 53 *ZmVQ* genes are shown in **Supplementary Table S4**, while the untreated seedlings were used as control. The expression level of most *ZmVQ* genes increased from 0 to 24 h after the NaCl stress treatment, and then decreased by 48 h after the treatment. In particular, the expression levels of *ZmVQ1*, *ZmVQ11*, *ZmVQ25*, *ZmVQ37*, *ZmVQ51*, and *ZmVQ52* were more than 20-times higher in drought-stressed seedlings than in control seedlings. By 24 h after the NaCl treatment, 36 out of the 53 *ZmVQ* genes were significantly up-regulated, while only 2 *ZmVQ* genes (ZmVQ16, 18) were down-regulated (**Figure 5**). By 48 h after the NaCl treatment, 29 out of 53 *ZmVQ* genes showed down-regulated expression, while four genes (*ZmVQ4*, *7*, *33*, *59*) showed up-regulated expression. These findings suggested that many *ZmVQ* genes were involved in the response to NaCl osmotic stress.

### Co-expression of *ZmVQ* and *ZmWRKY* Genes

**FIGURE 6:**
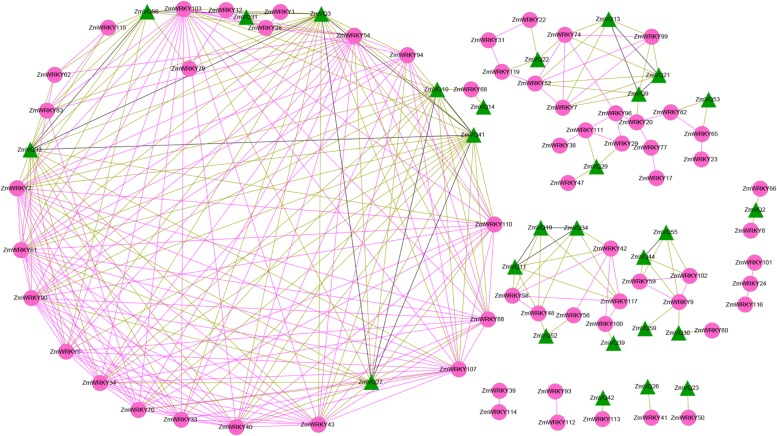
**Co-expression of *ZmVQ* and *ZmWRKY* genes.** Correlation coefficients of co-expression were >0.90. Red, green, and black lines indicate co-expression of two *ZmWRKY* genes, a *ZmVQ* gene and a *ZmWRKY* gene, and two *ZmVQ* genes, respectively. The circles with red color represent *ZmWRKY* genes, while the triangles with blue color represent *ZmVQ* genes.

To investigate the potential interactions between WRKYs and VQs in maize, we conducted co-expression analysis for the 61 *ZmVQ* and *ZmWRKY* genes identified in previous studies (**Figure 6**; **Supplementary Table S5**) using the RNA-seq data. Twenty-seven *ZmVQ* genes were co-expressed with 49 *ZmWRKY* genes (**Figure 6**). Among these 27 ZmVQ genes, 21 were co-expressed with more than one ZmWRKY gene, while six *ZmVQ* genes (*ZmVQ17*, *ZmVQ23*, *ZmVQ26*, *ZmVQ39*, *ZmVQ42*, and *ZmV*Q59) were co-expressed with only one *ZmWRKY* gene (**Figure 6**). Moreover, some *ZmVQ* (*ZmWRKY*) genes showed co-expression patterns with other members of *VQ* (*WRKY*) genes. Therefore, it is possible that the co-expression of VQ genes is required for the normal functions of *WRKY* genes. Further verification and functional analyses are needed to explore the physical interactions among these transcription factors in maize.

### Identification of *cis*-elements in *ZmVQ* and *ZmWRKY* Promoters

To explore the mechanisms by which these *VQ* genes responded to abiotic stresses, we searched for seven stress-related *cis*-acting regulatory elements (W-box, DRE, CG-box, MBS, ABRE, SARE, and G-box) in the *ZmVQ* and *ZmWRKY* promoters (2000-bp upstream sequences of start codons) (**Supplementary Figures S5** and **S6**). The results revealed that 41∼92% of ZmVQ and 32∼90% ZmWRKY promoter regions enriched the *cis*-elements (**Supplementary Figure S6**). More than four of these *cis*-elements were present in 42 (69%) *ZmVQ* and 71 (60%) *ZmWRKY* promoter sequences. Some elements were detected in more than one location in the promoter sequences. For example, the promoter of ZmVQ22 gene contained eight CG-box sequences. At least one W-box (which binds WRKY transcription factors during abiotic stress responses) was present in 91% (55 out of 61) of *ZmVQ* promoters and 42% (50 out of 119) of *ZmWRKY* promoters. **Supplementary Table S7** lists the *cis*-acting regulatory elements in the promoter regions of *ZmVQ* and *ZmWRKY* genes.

## Discussion

Proteins containing the VQ motif play vital roles in stress responses and seed development in plants. Functional and expression profile analyses of *VQ* genes could help us to understand their regulation during specific developmental processes and/or stress responses. In this study, we isolated and characterized 61 *ZmVQ* genes using the latest maize genome data. Our analyses showed that there are more *VQ* genes in maize than in rice (40 *OsVQ* genes) (Li et al., 2014b) and in *Arabidopsis* (34 *AtVQ* genes) (Cheng et al., 2012), but fewer *VQ* genes in maize than in soybean (74 *GmVQ* genes) (Wang et al., 2014). The maize genome is larger than the rice and *Arabidopsis* genomes. Therefore, it is plausible that the maize genome contains more *VQ* genes than do the rice and *Arabidopsis* genomes. The soybean genome is smaller than the maize genome, but it contains more *VQ* genes. This may be because of ancient genome duplication events. It was reported that the soybean genome underwent two duplications 59 and 13 million years ago, which resulted in multiple copies of around 75% of its genes (Schmutz et al., 2010). Consistent with this explanation, there are two or three copies of most *VQ* genes in the soybean genome (except for the single-copy gene *GmVQ55*) (Wang et al., 2014), while most of the *VQ* genes in the maize, rice, and *Arabidopsis* genomes are single-copy genes.

Intronless genes are very common in the genomes of higher eukaryotes (Louhichi et al., 2011). The gene structure analysis revealed that most of the *ZmVQ* genes are intronless. Only six genes have one or two introns (**Table 1**). In the phylogenetic analysis of *VQ* genes in maize, rice and *Arabidopsis*, the few VQ genes containing intron(s) were distributed in several different groups. This result suggested that these introns arose relatively recently and independently in maize, rice, and *Arabidopsis*. The *ZmVQ* genes identified in our study, along with those identified in other species, will be useful for further research on intron evolution in plants.

The majority (67.21%) of *ZmVQ* genes showed drought- and osmotic-responsive expression, similar to *VQ* genes in rice and *Arabidopsis*. In rice, 22 out of 39 *VQ* genes were induced by biotic and abiotic stresses (Kim et al., 2013). Specifically, *OsVQ2, 16, 20* were induced sharply by drought (Kim et al., 2013), as were their homologs in maize (*ZmVQ21, 13, 1*, **Figure 4**). Interestingly, *ZmVQ13* and *1* were significantly induced by NaCl treatment (**Figure 5**), suggesting that these two *ZmVQ* genes are involved in both drought and osmotic stress responses. In *Arabidopsis*, *AtVQ15* (also known as AtCaMBP25) and *AtVQ9* were shown to be involved in osmotic stress responses (NaCl and mannitol) (Perruc et al., 2004; Hu et al., 2013). Transgenic plants overexpressing *AtVQ15* showed increased sensitivity to osmotic stress induced by both NaCl and mannitol during the germination and seedling stages. In contrast, lines with down-regulated *AtVQ15* expression showed increased osmotic stress tolerance, compared with that of wild type (Perruc et al., 2004). *ZmVQ57*, the homolog of *AtVQ9*, was induced under both drought and osmotic stresses (**Figures 4** and **5**). *ZmVQ15*, *ZmVQ19*, and *ZmVQ28*, orthologs of *AtVQ15*, were transcriptionally induced under salt stress (**Figure 5**). Moreover, *ZmVQ19* was also induced by drought treatment (**Figure 4**), while *ZmVQ28* was up-regulated in drought-stressed leaf meristems and ovaries of 1DAP (Kakumanu et al., 2012). Stress-related *cis*-acting elements are important clues for how gene expression is regulated in response to environmental stimuli. We identified many *cis*-elements in the promoters of drought-responsive genes (**Supplementary Figure S7**; **Supplementary Table S8**). In other studies, expression analyses showed that these genes were induced by drought stress at the seedling stage (Zheng et al., 2010; Mao et al., 2015). In our study, most *ZmVQ* genes were responsive to drought and NaCl, and their promoters contained more than three *cis-*elements. It is possible that the homologous genes between maize and rice, and between maize and *Arabidopsis*, have the same or similar functions in response to abiotic stresses.

Recently, genome-wide expression analyses showed that some rice *VQ* genes were co-expressed with WRKY transcription factors during the responses to attacks by three different pathogens (Li et al., 2014b; Wang et al., 2015). Thus, VQ and WRKY proteins might assemble to form one complex to regulate the target gene. In the present study, we found that 27 *ZmVQ* genes were co-expressed with 49 *ZmWRKY* transcription factors under drought stress (**Figure 6**; **Supplementary Table S5**). Furthermore, some of the co-expressed *ZmWRKY* genes were shown to be involved in abiotic stresses (Wei et al., 2012), implying that some *VQ* and *WRKY* genes are involved in the same biological pathway. In our RNA-seq data, most of the ZmWRKY genes co-expressed with *ZmVQ* genes belonged to groups I and II (Dong et al., 2003; Wei et al., 2012), whereas several studied has reported that most *VQ* genes interact with *WRKY* genes of groups I and II in response to environmental stimuli (Rushton et al., 2010; Cheng et al., 2012; Wang et al., 2015). The results of the *cis*-element analysis indicated that 55 out of 61 *ZmVQ* genes (91%) contained a W-box motif (TTGAC[C/T], binding sites for WRKY transcription factor) in their 2.0-kb promoter regions, while a W-box was present in the 1.5-kb promoter regions of 14 out of 18 VvVQ genes (78%) in grapevine (Wang et al., 2015). The presence of W-boxes in the promoters of *ZmWRKY* genes suggested that there is feedback regulation among ZmWRKY members, as reported in *Arabidopsis* (Dong et al., 2003). This indicates that WRKY transcription factors might act as binding factors to regulate the expression of both *WRKY* and *VQ* genes, resulting in the expression of WRKY cofactors to ensure appropriate responses to environmental stimuli (Cheng et al., 2012). Our results have shown which WRKY proteins interact with which VQ proteins. Further research should explore their physical interactions during the responses to various abiotic stresses in maize, to provide further molecular evidence for these interactions in plants.

## Conclusion

We present here a comprehensive genome-wide study about the gene structure, phylogenetic relationship and tissue specificity of VQ domain-containing genes in maize. Expressional analysis of these VQ genes under drought and NaCl treatments suggested that some VQ genes are involved in abiotic stress responses. High co-expression correlation discovered between VQ genes and WRKY genes confirmed that many VQ genes and WRKY genes are likely functionally related. Information provided in this study will serve as a foundation for future exploration of the specific function of the VQ genes in the abiotic stress responses and their interaction with WRKY genes.

## Conflict of Interest Statement

The authors declare that the research was conducted in the absence of any commercial or financial relationships that could be construed as a potential conflict of interest. The reviewer Francisca Blanco and handling Editor Ariel Orellana declared that, despite having recently collaborated, the review process was conducted objectively.

## Footnotes

1 http://www.ncbi.nlm.nih.gov/

2 http://www.invitrogen.com/

3 www.illumina.com

4 www.promega.com

5 http://www.takara-bio.com

6 www.appliedbiosystems.com

## Supplementary Material

**TABLE S1 | Details of 78 different tissues used to generate RNA-seq data.**

**TABLE S2 | Premiers used for qPCR analyses of *ZmVQ* genes.**

**TABLE S3 | Accession number of *ZmWRKY* genes.**

**TABLE S4 | Transcript abundance (qPCR values) of genes in maize seedlings under NaCl stress.**

**TABLE S5 | The coefficient values of *ZmVQ* and *ZmWRKY* genes.**

**TABLE S6 | Stress-related *cis*-elements in promoters of *ZmVQ* and *ZmWRKY* genes.**

**TABLE S7 | Transcript abundance (qPCR values) of *ZmVQ* genes in seven maize different tissues.**

**TABLE S8 | Stress-related *cis*-elements in promoters of drought- responsive marker genes.**
