# Supplementary material for: Genome-Wide Identification of VQ Motif-Containing Proteins and their Expression Profiles Under Abiotic Stresses in Maize
Source: Front Plant Sci. 2016 Jan 5;6:1177. doi: 10.3389/fpls.2015.01177 (PMC4700186; doi:10.3389/fpls.2015.01177)
Supplement: Supplementary file 9 [file Image_1.PDF]

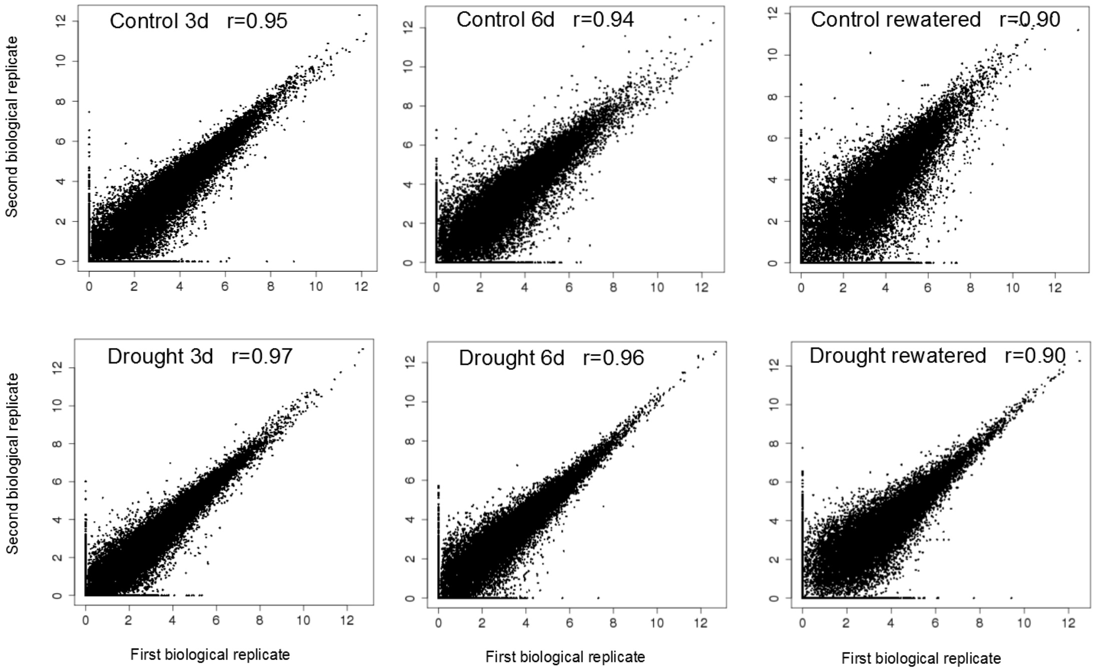

Supplementary Figure S1. Correlation analysis between two biological replicates. we calculated the expression level (FPKM) for each replicate. The normalized data of log2 (FPKM +1) was used to calculate the Pearson Correlation Coefficient (PCC). The average R^2 for the biological replicates was 0.94.

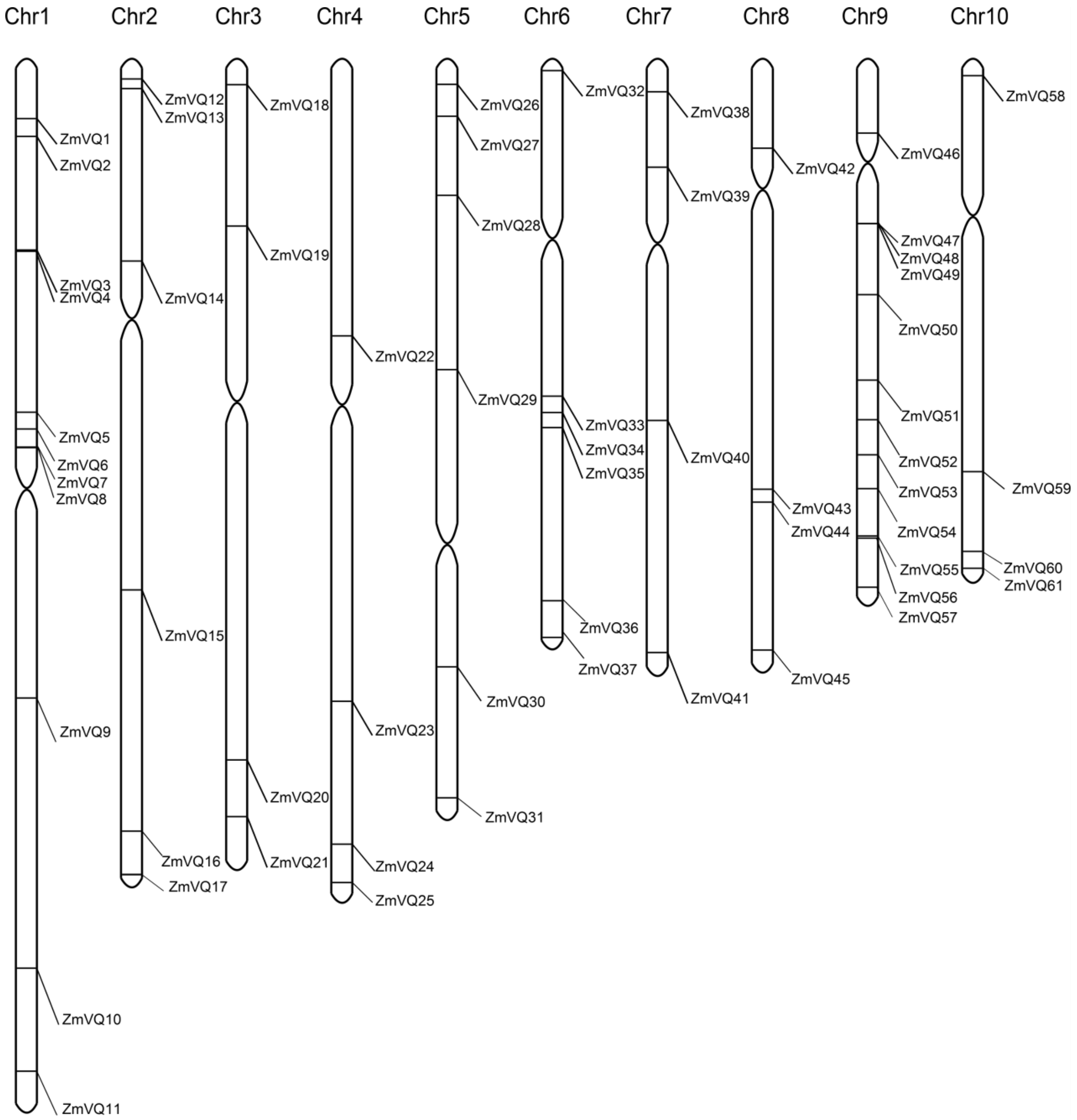

Supplementary Figure 2. Distribution of *ZmVQ* genes on ten chromosomes. The 61 ZmVQ genes were mapped to the 10 chromosome in maize. The physical position was labeled by the black line and their genes name.

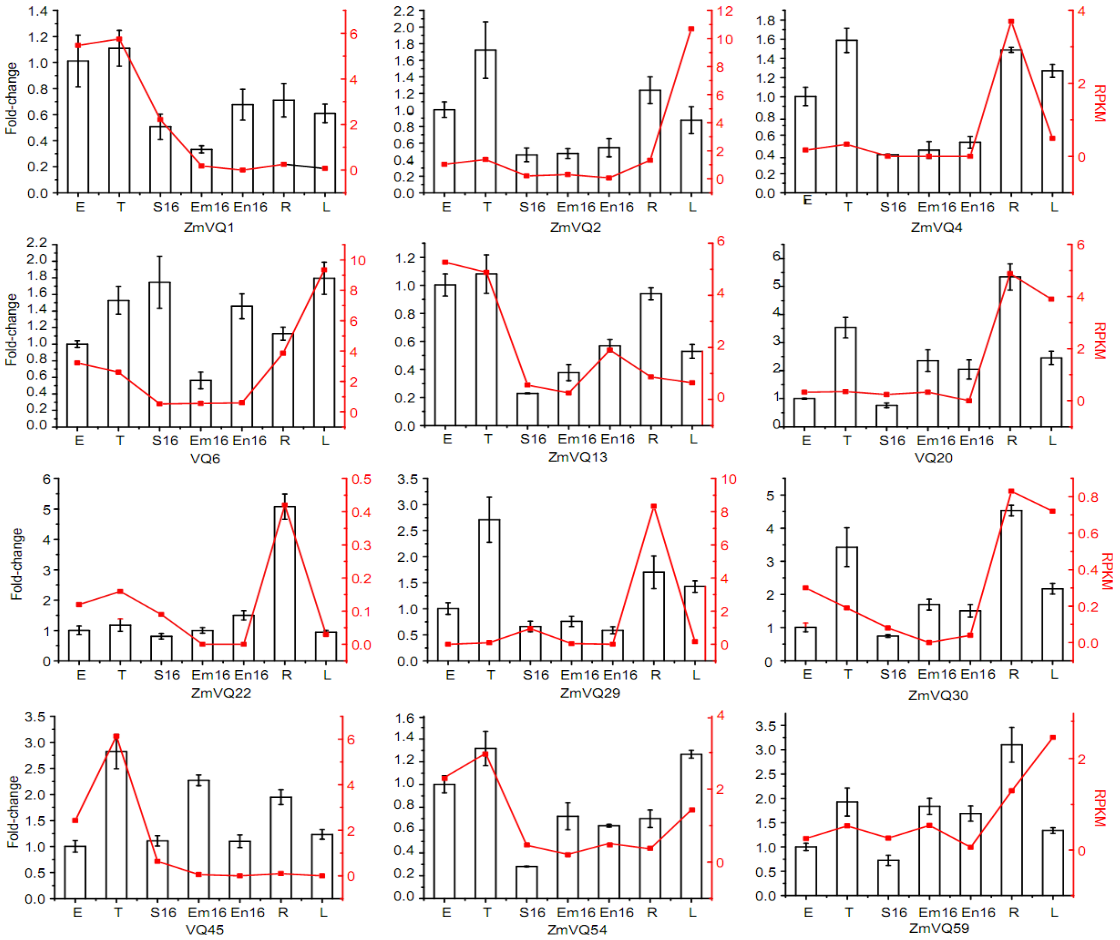

Supplementary Figure S3 qRT-PCR validation of the relative expression levels among ZmVQ gene in seven different tissues.

The relative expression levels of ZmVQ genes were determined by qRT-PCR (white bars) in ear (E), tassel (T), seed of 16-days after pollination (S16), embryo of 16-days after pollination (Em16), endosperm of 16-days after pollination (En16), root of two-week-old (R) and leaf of 2-week-old (L), respectively. Three independent experiments were done with similar results, each with three biological repeats. Values are means+SE (n=3). The red lines at the right border of the figure indicate mRNA abundance (RPKM) according to RNA-seq data, and black lines at the left side of the figure indicate the relative expression levels calculated by qRT-PCR.

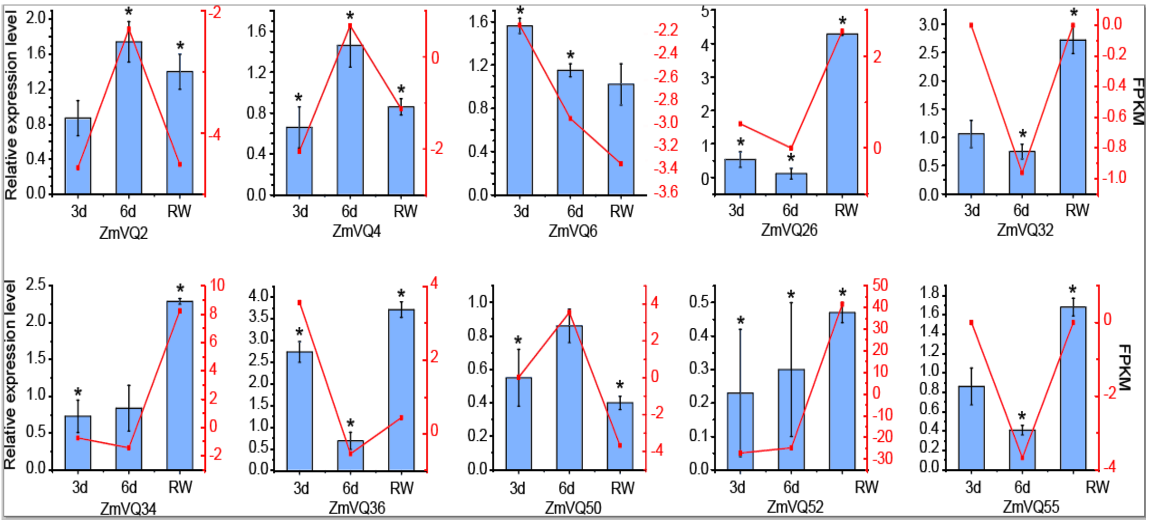

Supplementary Figure S4 qRT-PCR validation of relative expression levels of ZmVQ genes in plants under drought stress

The expression levels in the controls for 3 days, 6 days, and re-watering were normalized to a value of 1, which were not shown in the figure. The relative expression levels to the corresponding controls of ZmVQ genes were determined by qRT-PCR (blue bars) indicated by the black lines (left side of the figure). Values were means+SD (n=3). The relative expression levels to the controls of ZmVQ genes were shown as FPKM values indicated by red lines (right side of the figure). 3d, 6d and RW represent the time points of drought for 3 days, 6 days, and re-watering (the seventh day) , respectively. The significant differences are noted by an asterisk.

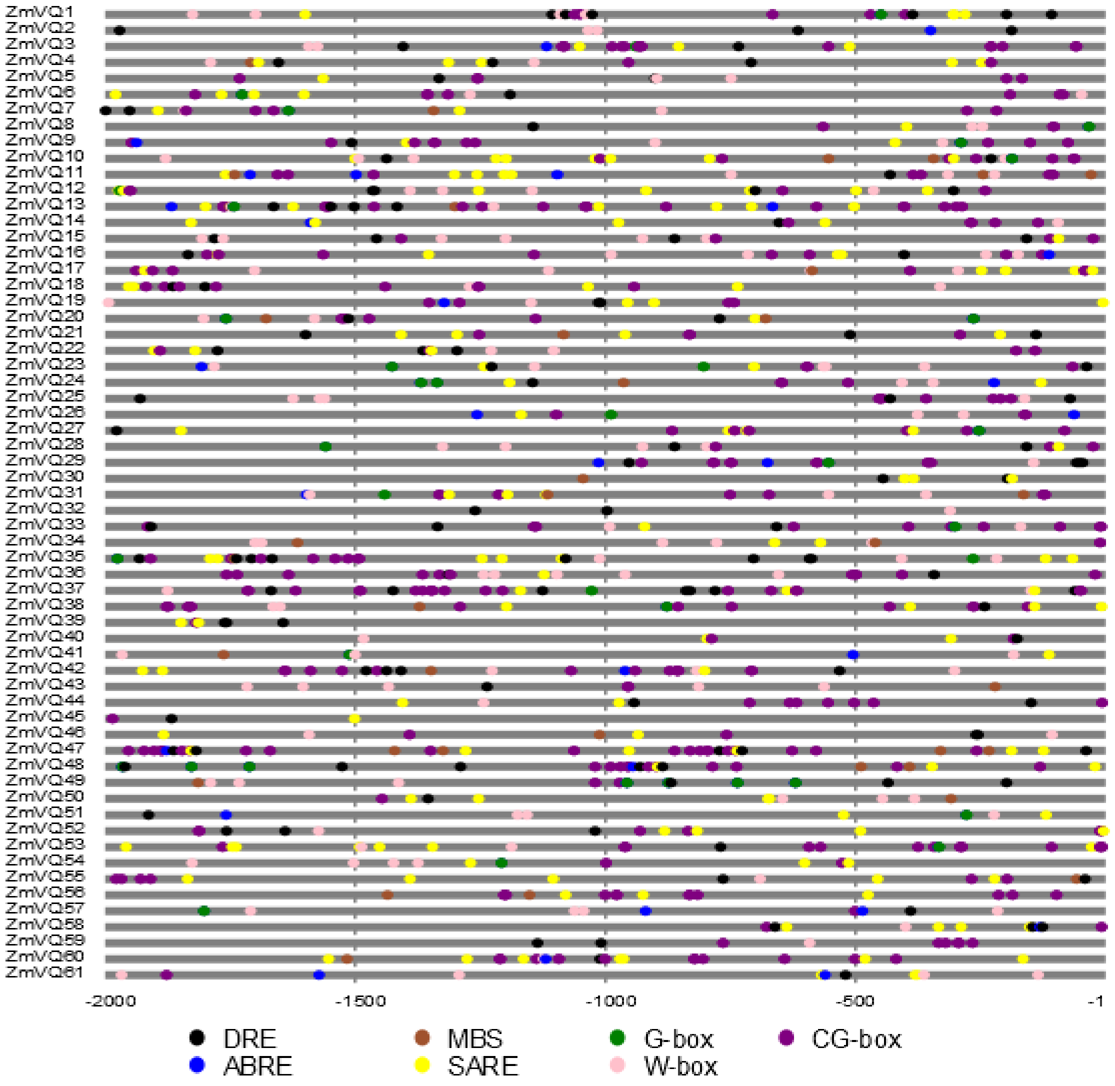

Supplementary Figure S5. Distribution of stress-related cis-elements in promoters of ZmVQ genes. The promoter sequences (-2000bp genomic DNA sequences upstream of the start codon) of ZmVQ genes were labeled by the seven cis-acting regulatory elements with different colored circles at the bottom of this figure.

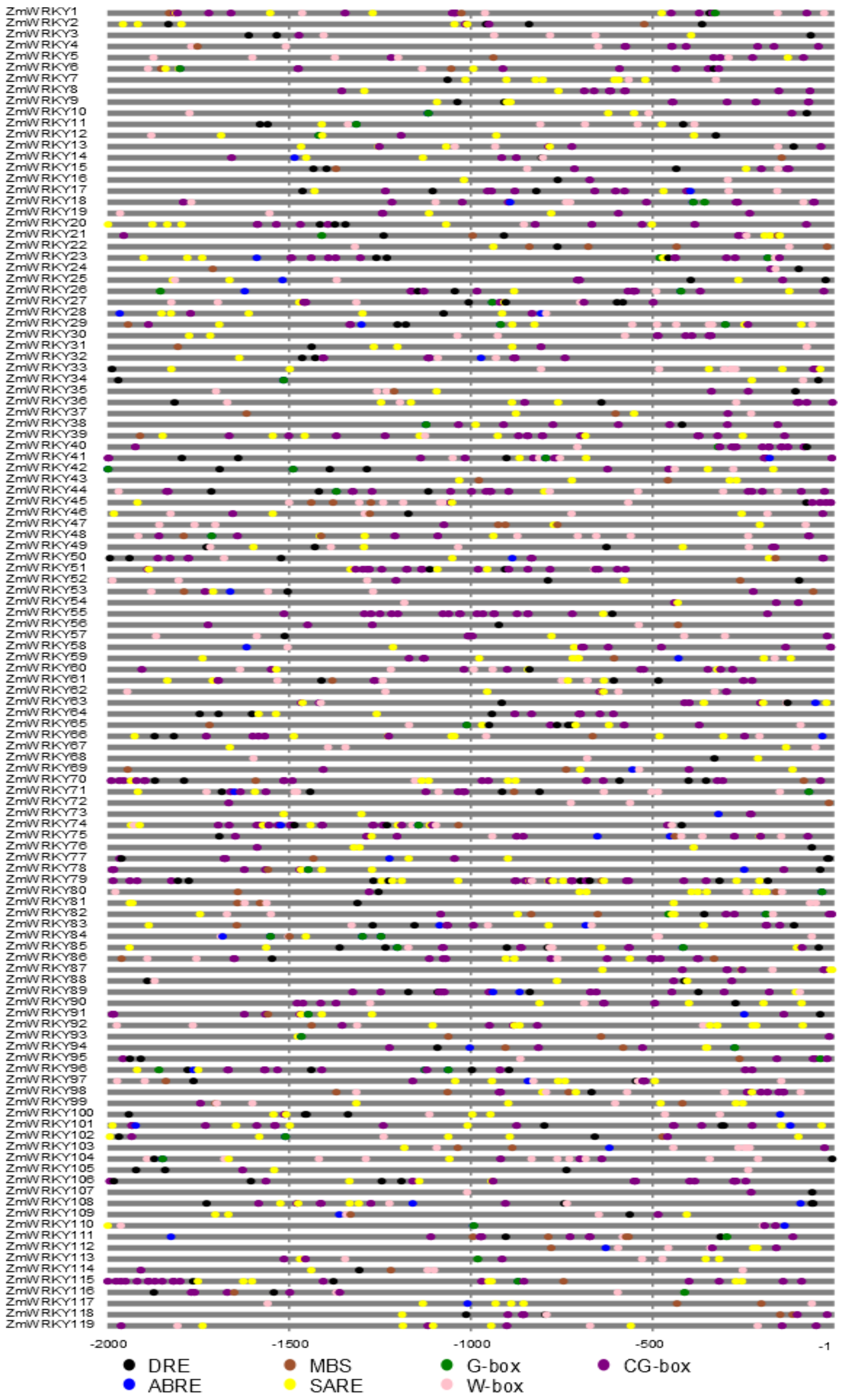

Supplementary Figure S6. Distribution of stress-related cis-elements in promoters of ZmWRKY genes. The promoter sequences (-2000bp genomic DNA sequences upstream of the start codon) of ZmWRKY genes were labeled by the seven cis-acting regulatory elements with different colored circles at the bottom of this figure.

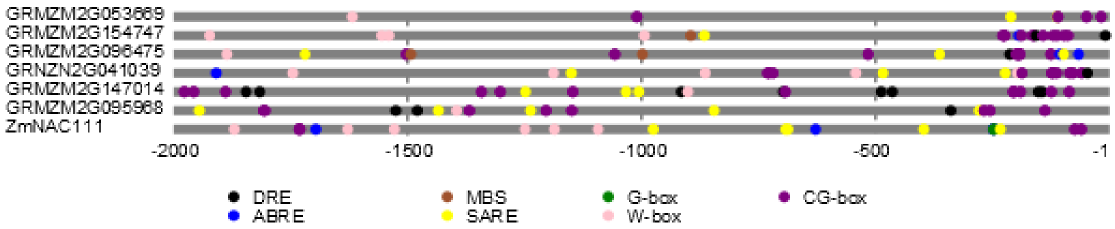

Supplementary Figure S7. Distribution of stress-related cis-elements in promoters of drought -responsive marker genes. The promoter sequences (-2000bp genomic DNA sequences upstream of the start codon) of the drought responsive genes were labeled by the seven cis-acting regulatory elements with different colored circles at the bottom of this figure. The marker genes for responses to drought stress cited from the references below.

1) Mao H., Wang H., Liu S., Li Z., Yang X., Yan J., et al. (2015). A transposable element in a NAC gene is associated with drought tolerance in maize seedlings. Nat Commun 6: 8326.
2) Zheng J., Fu J., Gou M., Huai J., Liu Y., Jian M., et al. (2010). Genome-wide transcriptome analysis of two maize inbred lines under drought stress. Plant Mol Biol 72: 407-21.
